# Existence Theorems for Vector Equilibrium Problems via Quasi-Relative Interior

**DOI:** 10.1155/2013/150130

**Published:** 2013-05-16

**Authors:** Capătă Adela Elisabeta

**Affiliations:** Technical University of Cluj-Napoca, Department of Mathematics, Cluj-Napoca, G. Bariţiu 25-28, 400027 Jud. Cluj, Romania

## Abstract

The aim of this paper is to present new existence theorems for solutions of vector equilibrium problems, by using weak interior type conditions and weak convexity assumptions.

## 1. Introduction

Starting with the paper of Blum and Oettli [1], the field of equilibrium problems is intensively studied by researchers, and many papers dealing with vector equilibrium problems were written. The interest of the researchers on this topic is due to the fact that equilibrium problems represent a natural and unified framework for other problems, such as optimization problems, variational inequality problems, and saddle-point problems, problems which until now were separately studied and have applications in physics, economics, and so forth, (see, for instance, [2]). Since then, a large variety of vector equilibrium problems were considered and the authors studied the existence of solutions (see, for instance, [3–10]), well posedness (see, for instance, [11, 12]), and sensitivity analysis (see, for instance, [13, 14]).

Most of the existence results are based on the hypothesis of nonemptyness of the ordering cone. In this paper, based on weak convexity assumptions defined by the means of quasi-relative interior of a convex set introduced in [15] and by using a quite recent separation theorem, whose statement was given in [16], we establish existence theorems for solutions of vector equilibrium problems. Then, the results are applied to vector optimization problems and to vector variational inequalities.

The paper is organized as follows. In Section 2 we recall some notions and auxiliary results that we need throughout this paper. Then, in Section 3 we present the main results of this paper, whose statements are given in the terms of quasi-relative interior and weak convexity assumptions. These results are applied to a vector optimization problem and to vector variational inequalities in Sections 4 and 5, respectively.

## 2. Preliminaries

Let *Y* be a separated locally convex space, and let *C*⊆*Y* be a nontrivial convex cone. Having Y*, the dual space of *Y*, the dual cone of *C* is

(1) $$\begin{array}{c} C^{*}:=\left\{y^{*}\in Y^{*}\;|\;y^{*}\left(c\right)\geq 0,\forall c\in C\right\}. \end{array}$$

We recall the definitions of quasi-interior and quasi-relative interior of a convex set and some useful properties of the quasi-relative interior notion.

**Definition 1**

Let *M* be a nonempty convex subset of *Y*. The quasi interior of *M* is the set

(2) $$\begin{array}{c} \text{qi}\;M:=\left\{y\in M\;|\;\mathrm{cl}\;\mathrm{cone}\left(M-y\right)=Y\right\}. \end{array}$$

**Definition 2 (see [17])**

Let *M* be a nonempty convex subset of *Y*. The quasi-relative interior of *M* is the set

(3) qri  M :={y∈M ∣ cl⁡ cone⁡(M−y)is  a  linear  subspace  of  Y}.

In a separable Banach space the quasi-relative interior of any nonempty closed convex set is nonempty (cf. [17]). For the proof of the following properties and other useful properties of the quasi-relative interior of a convex set, we refer the reader to [17, 18], respectively.

**Proposition 3**

Let *M* and *N* be nonempty convex subsets of *Y*, *y* ∈ *Y*, and *α* ∈ ℝ. Then the following statements are true:

- qri  *M*+ qri  *N*⊆ qri  (*M* + *N*);
- qri  (*αM*) = *α* qri  *M*;
- qri  ( qri  *M*) = qri  *M*;
- qri  (*M* − *y*) = qri  *M* − *y*;
- *If * qri  *M* ≠ *∅*, *then *
- cl⁡  qri  *M* = cl⁡  *M*;
- cl⁡ cone⁡(qri  *M*) = cl⁡ cone⁡  *M*.

The normal cone of a convex subset *M* of *Y* at *y*_0_ ∈ *Y* is defined as

(4) $$\begin{array}{c} N_{M}\left(y_{0}\right):=\left\{y^{*}\in Y^{*}\;|\;y^{*}\left(y-y_{0}\right)\leq 0,\forall y\in M\right\}. \end{array}$$

By means of the normal cone, the next characterizations of the abovementioned interior notions hold, and they can be found in [16, 17].

**Theorem 4**

Let *M* be a nonempty convex subset of  *Y* and *y* ∈ *M*. Then *y*∈qi  *M* if and only if *N*_*M*_(*y*) = {0}.

**Theorem 5**

Let *M* be a nonempty convex subset of  *Y* and *y* ∈ *M*. Then *y* ∈ qri  *M* if and only if *N*_*M*_(*y*) is a linear subspace of Y*.

For other generalizations of the classical interior we refer the reader to [19–22]. Whenever the interior of the set *M* is nonempty, then int  *M* = qri  *M* (see [17, Corollary 2.14]). If *Y* is finite dimensional, then qri  *M* = ri  *M* (see [17]), where by ri  *M* we understand the relative interior of *M*, that is, the interior with respect to the affine hull.

The following characterization for the quasi-interior of a convex cone holds, and it can be found in [23].

**Proposition 6**

Let *C* be a convex cone in a locally convex space *Y*, and let *c* ∈ *C*. Then *c* ∈ qi  *C* if and only if

(5) $$\begin{array}{c} y^{*}\left(c\right)>0\;\forall y^{*}\in C^{*}\setminus \left\{0\right\}. \end{array}$$

**Lemma 7 (see [17])**

Let *C* be a convex cone in a separable locally convex space *Y*. Then *c* ∈ qi  *C* if and only if *c*∈qri*C* and cl⁡  (*C* − *C*) = *Y*.

**Lemma 8 (see [17])**

Let *C* be a convex cone in a locally convex space *Y*. Then

(6) $$\begin{array}{c} tC+\left(1-t\right)\text{ qri }\;C\subseteq \text{ qri }\;C \end{array}$$

for all *t* ∈ [0,1).

The statement of the next theorem is due to [16], where it was proved for normed spaces, and, later on, it was proved for separated locally convex spaces by [24].

**Theorem 9**

Let *M* be a nonempty convex subset of *Y* and *y*_0_ ∈ *M*∖qri*M*. Then, there exists y* ∈ Y*∖{0} such that y*(*y*) ≥ y*(*y*_0_) for all *y* ∈ *M*.

For other separation theorems which involve the quasi-relative interior we refer the reader to [25].

**Definition 10**

A function *f* : *A* → *Y* is said to be generalized *C*-subconvexlike on *A* if cone   *f*(*A*) + qri *C* is convex.

**Proposition 11**

If *f*(*A*) + *C* is convex, then cone  *f*(*A*) + qri*C* is also convex.

**Proof**

Let *z*_1_, *z*_2_ ∈ cone⁡ *f*(*A*) + qri  *C* and let *t* ∈ (0,1). So, there exist *t*_1_ ≥ 0, *t*_2_ ≥ 0, *a*_1_, *a*_2_ ∈ *A*, and *c*_1_, *c*_2_ ∈ qri  *C* such that

(7) $$\begin{array}{c} z_{1}=t_{1}f\left(a_{1}\right)+c_{1},\;\;\;z_{2}=t_{2}f\left(a_{2}\right)+c_{2}. \end{array}$$

*Case *1. If *t*_1_ = 0 and *t*_2_ = 0, then *z*_1_ = *c*_1_, *z*_2_ = *c*_2_ and

(8) tz1+(1−t)z2  =tc1+(1−t)c2∈qri  C⊆cone⁡  f(A)+qri  C.

*Case *2. If *t*_1_ ≠ 0 or *t*_2_ ≠ 0, denote

(9) $$\begin{array}{c} t_{1}^{\prime }=\frac{t_{1}}{t_{1}+t_{2}}\in \left[0,1\right],\;\;\;t_{2}^{\prime }=\frac{t_{2}}{t_{1}+t_{2}}\in \left[0,1\right],\\ c_{1}^{\prime }=\frac{c_{1}}{t_{1}+t_{2}}\in \text{qri}\;C,\;\;c_{2}^{\prime }=\frac{c_{2}}{c_{1}+c_{2}}\in \text{qri}\;C,\\ z_{1}^{\prime }=\frac{z_{1}}{t_{1}+t_{2}},\;\;z_{2}^{\prime }=\frac{z_{2}}{t_{1}+t_{2}}. \end{array}$$

So, we have

(10) $$\begin{array}{c} z_{1}^{\prime }=t_{1}^{\prime }f\left(a_{1}\right)+c_{1}^{\prime },\;z_{2}^{\prime }=t_{2}^{\prime }f\left(a_{2}\right)+c_{2}^{\prime }. \end{array}$$

In what follows we will prove that *tz*_1_′ + (1 − *t*)*z*_2_′ ∈ cone⁡ *f*(*A*) + qri  *C*. For this, we evaluate

(11) $$\begin{array}{c} tz_{1}^{\prime }+\left(1-t\right)z_{2}^{\prime }=tt_{1}^{\prime }f\left(a_{1}\right)+tc_{1}^{\prime }+\left(1-t\right)t_{2}^{\prime }f\left(a_{2}\right)+\left(1-t\right)c_{2}^{\prime }. \end{array}$$

Dividing this equality by the positive number *tt*_1_′ + (1 − *t*)*t*_2_′ it yields

(12) tz1′+(1−t)z2′tt1′+(1−t)t2′ =tt1′tt1′+(1−t)t2′f(a1)+(1−t)t2′tt1′+(1−t)t2′f(a2)  +ttt1′+(1−t)t2′c1′+1−ttt1′+(1−t)t2′c2′ =tt1′tt1′+(1−t)t2′[f(a1)+c1′]+(1−t)t2′tt1′+(1−t)t2′[f(a2)+c2′]  +t(1−t1′)tt1′+(1−t)t2′c1′+(1−t)(1−t2′)tt1′+(1−t)t2′c2′.

By the above equalities and the convexity of *f*(*A*) + *C*, we have

(13) tz1′+(1−t)z2′tt1′+(1−t)t2′∈f(A)+C+tqri  C+(1−t)qri  C⊆f(A)+C+qri  C⊆f(A)+qri  C.

Thus,

(14) $$\begin{array}{c} tz_{1}^{\prime }+\left(1-t\right)z_{2}^{\prime }\in \mathrm{cone}\;f\left(A\right)+\text{qri}\;C, \end{array}$$

whence multiplying this relation by *t*_1_ + *t*_2_ we obtain the conclusion.

## 3. Existence Results

Throughout this paper we study the following strong vector equilibrium problem:

$$\begin{array}{cc} \left(\text{VEP}\right) & \;\text{find}\;a\in A\;\text{such}\;\text{that}\;f\left(a,b\right)\notin -C\setminus \left\{0\right\}\;\forall b\in B, \end{array}$$

where *A* and *B* are non-empty sets, *f* : *A* × *B* → *Y*, and *C* has a non-empty quasi-relative interior.

**Definition 12**

We say that a point *a*_0_ ∈ *A* is a quasi-relative solution of ((VEP)) if

(15) $$\begin{array}{c} f\left(a_{0},b\right)\notin -\text{qri}\;C\;\forall b\in B. \end{array}$$

**Theorem 13**

Suppose that the following conditions are satisfied:

- for every *b* ∈ *B*, the function *b* ↦ *f*(·, *b*) is generalized *C*-subconvexlike on *A*;
- cl⁡  (*C* − *C*) = *Y*;
- for every *b* ∈ *B*, cl⁡ cone⁡(cone⁡  *f*(*A*, *b*) + qri  *C*) is not a linear subspace of *Y*.

Then, problem ((VEP)) admits a quasi-relative solution.

**Proof**

Suppose by contradiction that ((VEP)) has no solutions; that is, for any *a* ∈ *A* there exists *b*_0_ ∈ *B* such that

(16) $$\begin{array}{c} f\left(a,b_{0}\right)\in -\text{qri}\;C. \end{array}$$

This leads us to

(17) $$\begin{array}{c} 0\in f\left(a,b_{0}\right)+\text{qri}\;C, \end{array}$$

which implies that

(18) $$\begin{array}{c} 0\in \mathrm{cone}\;f\left(A,b_{0}\right)+\text{qri}\;C. \end{array}$$

Assumption (i) assures the convexity of the set cone⁡  *f*(*A*, *b*_0_) + qri  *C*, and, together with the relation (18) and assumption (iii), it gives

(19) $$\begin{array}{c} 0\notin \text{qri}\;\left(\mathrm{cone}\;f\left(A,b_{0}\right)+\text{qri}\;C\right). \end{array}$$

The separation Theorem 9 of convex sets assures the existence of a nonzero functional y* ∈ Y* such that

(20) $$\begin{array}{c} y^{*}\left(y\right)\geq y^{*}\left(0\right)\;\forall y\in \mathrm{cone}\;f\left(A,b_{0}\right)+\text{qri}\;C. \end{array}$$

So

(21) $$\begin{array}{c} y^{*}\left(tf\left(a,b_{0}\right)+c\right)\geq 0\;\forall t\geq 0,\;\;a\in A,\;\;c\in \text{qri}\;C. \end{array}$$

Taking *t* = 0, by cl⁡(qri  *C*) = cl⁡ *C* and (21) we have

(22) $$\begin{array}{c} y^{*}\left(c\right)\geq 0\;\forall c\in \text{C}, \end{array}$$

which gives

(23) $$\begin{array}{c} y^{*}\in C^{*}. \end{array}$$

Further, for any *t* > 0, dividing by *t* relation (21) it implies

(24) $$\begin{array}{c} y^{*}\left(f\left(a,b_{0}\right)+\frac{c}{t}\right)\geq 0\;\text{for}\;\text{any}\;a\in A,\;\;c\in \text{qri}\;C. \end{array}$$

Now letting *t* → *∞* in the above relation we get

(25) $$\begin{array}{c} y^{*}\left(f\left(a,b_{0}\right)\right)\geq 0\;\forall a\in A. \end{array}$$

Since *f*(*a*, *b*_0_)∈−qri  *C*, by assumption (ii), Lemma 7, and Proposition 6, we get

(26) $$\begin{array}{c} y^{*}\left(f\left(a,b_{0}\right)\right)<0, \end{array}$$

which is a contradiction to (25), and this completes the proof.

This theorem allows us to obtain existence results for important practical spaces, whose ordering cones have empty interiors, but nonempty quasi-relative interiors. This is the case of the Banach space *L* ^*P*^(*T*, *μ*) with the positive cone

(27) $$\begin{array}{c} L_{+}^{p}\left(T,\mu \right)=\left\{u\in L^{p}\left(T,\mu \right)\;|\;u\left(t\right)\geq 0\;\text{a}.\text{e}.\;\text{in}\;\left[0,T\right]\right\}, \end{array}$$

where *T* > 0 is a real constant, [0, *T*] is an interval, (*T*, *μ*) is a *σ*-finite measure space, and *p* ∈ [1, *∞*).

The next result deals with stronger assumptions than the ones presented in Theorem 13.

**Corollary 14**

Suppose that the following conditions are satisfied:

- for every *b* ∈ *B*, the function *b* ↦ *f*(·, *b*) is generalized *C*-subconvexlike on *A*;
- cl⁡ (*C* − *C*) = *Y*;
- for every *b* ∈ *B*, 0 ∉ qri[cone⁡(cone⁡*f*(*A*, *b*) + qri*C*)].

Then, problem ((VEP)) admits a quasi-relative solution.

**Proof**

Since 0 ∉ qri  [cone⁡(cone⁡*f*(*A*, *b*) + qri  *C*)] and

(28) $$\begin{array}{c} 0\in \mathrm{cone}\left(\mathrm{cone}f\left(A,b\right)+\text{qri}\;C\right) \end{array}$$

for every *b* ∈ *B*, by the definition of the quasi-relative interior, we get

(29) cl⁡ cone⁡[cone⁡(cone⁡f(A,b)+qri  C)]  =cl⁡ cone⁡(cone⁡f(A,b)+qri  C)

is not a linear subspace of *Y*. So all the assumption of Theorem 13 are satisfied, and the conclusion follows now by this theorem.

**Remark 15**

In Theorem 13 and Corollary 14, according to Proposition 11, the generalized *C*-subconvexlike assumption of the function *b* ↦ *f*(·, *b*) on *A* can be replaced by the convexity of the set *f*(*A*, *b*) + *C*.

## 4. Existence Results for Vector Optimization Problems

Let *B* = *A*, and let the function *F* : *A* → *Y*. In this section we study the vector optimization problem,

(VOP)  find  a∈A  such  that  F(b)−F(a)∉−C∖{0}∀b∈A.

According to [26], the point *F*(*a*) is called quasi-relative minimal point of the set *F*(*A*), that is,

(30) $$\begin{array}{c} F\left(A\right)-F\left(a\right)\notin -\text{qri}\;C, \end{array}$$

while *a* is a quasi-relative minimizer of ((VOP)), that is,

(31) $$\begin{array}{c} F\left(b\right)-F\left(a\right)\notin -\text{qri}\;C\;\forall b\in A. \end{array}$$

By Theorem 13 and Corollary 14 we have the following results.

**Theorem 16**

Suppose that the following conditions are satisfied:

- for every *b* ∈ *A*, the function *F*(*b*) − *F*(·) is generalized *C*-subconvexlike on *A*;
- cl⁡(*C* − *C*) = *Y*;
- for every *b* ∈ *A*, cl⁡  cone⁡(cone⁡(*F*(*b*) − *F*(*A*)) + qri*C*) is not a linear subspace of *Y*.

Then, problem ((VOP)) admits a quasi-relative solution.

**Proof**

Define the function *f* : *A* × *A* → *Y* by

(32) $$\begin{array}{c} f\left(a,b\right)=F\left(b\right)-F\left(a\right)\;\forall a,\;\;b\in A. \end{array}$$

It is easy to see that all the assumptions of the Theorem 13 are satisfied by this function *f*. So, problem ((VEP)) admits a solution, which implies that problem ((VOP)) has a solution, and the proof is completed.

**Corollary 17**

Suppose that the following conditions are satisfied:

- for every *b* ∈ *A*, the function *b* ↦ *F*(*b*) − *F*(·) is generalized *C* subconvexlike on *A*;
- cl⁡(*C* − *C*) = *Y*;
- for every *b* ∈ *A*, 0 ∉ qri[cone⁡(cone⁡(*F*(*b*) − *F*(*A*)) + qri*C*)].

Then, problem ((VOP)) admits a quasi-relative solution.

To show that the set of functions which satisfies the assumptions of Corollary 17 is nonempty, we give the following example.

**Example 18**

Let *A* = [0,1], *Y* = ℝ^2^, *C* = ℝ_+_^2^, and *F*(*a*) = (*a*, 0). We have that

(33) $$\begin{array}{c} F\left(b\right)-F\left(a\right)=\left(b-a,0\right)\;\forall a,b\in \left[0,1\right]. \end{array}$$

Since *F*(*b*) − *F*(*A*) + *C* = (*b* − *A*, 0) + *C* and *A* is a convex set, we deduce that

(34) $$\begin{array}{c} F\left(b\right)-F\left(A\right)+C \end{array}$$

is also convex and that, by Proposition 11, the first assumption of Corollary 17 is verified. The second assumption is obviously satisfied, and we still have to verify its third assumption. Because int⁡  *C* ≠ *∅*, then it is equal to qri  *C*. The set

(35) int⁡[cone⁡(cone⁡(F(b)−F(A))+int⁡  C)]=int⁡[ℝ×[0,∞)]=ℝ×(0,∞),

for all *b* ∈ (0,1], while for *b* = 1

(36) $$\begin{array}{c} \mathrm{int}\left[\mathrm{cone}\left(\mathrm{cone}\left(F\left(b\right)-F\left(A\right)\right)+\mathrm{int}\;C\right)\right]=\left(0,\infty \right)\times \left(0,\infty \right). \end{array}$$

Thus,

(37) $$\begin{array}{c} \left(0,0\right)\notin \mathrm{int}\left[\mathrm{cone}\left(\mathrm{cone}\left(F\left(b\right)-F\left(A\right)\right)+\mathrm{int}\;C\right)\right]\;\forall b\in A, \end{array}$$

and assumption (iii) is checked.

## 5. Existence Results for Vector Variational Inequalities 

Let *A* be a non-empty convex subset of a vector space, let *B* = *A*, and let the operator *F* : *A* → *ℒ*(*X*, *Y*), where *ℒ*(*X*, *Y*) denotes the set of all linear and continuous functions defined on *X* with values on *Y*. Throughout this section we study the Minty vector variational inequality:

(MVI)  find  a∈A  such  that  〈F(b),b−a〉∉−C∖{0}∀b∈A.

**Definition 19**

We say that a point *a*_0_ ∈ *A* is a quasi-relative solution of ((MVI)) if

(38) $$\begin{array}{c} \langle F\left(b\right),b-a_{0}\rangle \notin -\text{qri}\;C\;\forall b\in A. \end{array}$$

By Theorem 13 and Corollary 14 we have the following results.

**Theorem 20**

Suppose that the following conditions are satisfied:

- cl⁡  (*C* − *C*) = *Y*;
- for every *b* ∈ *A*, cl⁡  cone⁡(cone⁡〈*F*(*b*), *b* − *A*〉+qri*C*) is not a linear subspace of *Y*.

Then, problem ((MVI)) admits a quasi-relative solution.

**Proof**

Let the function *f* : *A* × *A* → *Y* be defined by

(39) $$\begin{array}{c} f\left(a,b\right)=\langle F\left(b\right),b-a\rangle \;\forall a,b\in A. \end{array}$$

Obviously *f* fulfills assumptions (ii) and (iii) of Theorem 13. It remains to show that the first assumption of this theorem is also verified by this function *f*.

Since *A* is a convex set, by the definition of the function *f* we deduce that *f*(*A*, *b*) is a convex set for every *b* ∈ *A*. By this we get

(40) $$\begin{array}{c} f\left(A,b\right)+C \end{array}$$

convex, which together with Proposition 11 gives the conclusion, and proof is completed.

**Corollary 21**

Suppose that the following conditions are satisfied:

- cl⁡  (*C* − *C*) = *Y*;
- for every *b* ∈ *A*, 0 ∉ qri[cone⁡(cone⁡〈*F*(*b*), *b* − *A*〉+qri*C*)].

Then, problem ((MVI)) admits a quasi-relative solution.

In what follows we turn our attention to existence results for the Stampacchia vector variational inequality

(SVI)  find  a0∈A  such  that  〈F(a),b−a〉∉−C∖{0}∀b∈A.

**Definition 22**

We say that a point *a*_0_ ∈ *A* is a quasi-relative solution of ((SVI)) if

(41) $$\begin{array}{c} \langle F\left(a_{0}\right),b-a_{0}\rangle \notin -\text{qri}\;C\;\forall b\in A. \end{array}$$

Next we recall a definition concerning vector variational inequalities (see [27]).

**Definition 23**

Let c* ∈ C*∖{0}. The operator *F* is said to be c*-upper hemicontinuous if *A* is a convex set, and, for all *a*, *b* ∈ *A* the function

(42) $$\begin{array}{c} \forall \lambda \in \left[0,1\right]\mapsto c^{*}(\langle F\left(\lambda b+\left(1-\lambda \right)a\right),b-a)\rangle \in \mathbb{R} \end{array}$$

is upper semicontinuous at 0.

**Theorem 24**

Let c* ∈ C*∖{0}, let *a*_0_ ∈ *A*, and suppose that the following conditions are satisfied:

- c*(〈*F*(*b*), *b* − *a*_0_〉+qri*C*) ≥ 0 for all *b* ∈ *A*;
- *F* is c*-upper hemicontinuous.

Then, problem ((SVI)) admits a quasi-relative solution.

**Proof**

By assumption (i)

(43) $$\begin{array}{c} c^{*}\left(\langle F\left(b\right),b-a_{0}\rangle +\text{qri}\;C\right)\geq 0\;\forall b\in A, \end{array}$$

whence, by using Lemma 8, we deduce that

(44) $$\begin{array}{c} \langle F\left(b\right),b-a_{0}\rangle \notin -C\setminus \left\{0\right\}-\text{qri}\;C\subseteq -\text{qri}\;C\;\forall b\in A, \end{array}$$

that is, *a*_0_ is a quasi-relative solution of ((MVI)). In what follows we will prove that *a*_0_ is also a quasi-relative solution of ((SVI)).

Because *A* is convex, then

(45) $$\begin{array}{c} \lambda b+\left(1-\lambda \right)a_{0}\in A, \end{array}$$

for all *b* ∈ *A* and all *λ* ∈ [0,1], and by (43), we get

(46) $$\begin{array}{c} c^{*}\left(\langle F\left(\lambda b+\left(1-\lambda \right)a_{0}\right),b-a_{0}\rangle +\text{qri}\;C\right)\geq 0\;\forall \lambda \in \left[0,1\right]. \end{array}$$

For each *ϵ* > 0, there exists, according to (ii), a number *λ* ∈ (0,1] such that

(47) c∗(〈F(λb+(1−λ)a0),b−a0〉+qri  C)  <c∗(〈F(a0),b−a0〉+qri  C)+ϵ.

This, together with (46), gives

(48) $$\begin{array}{c} 0<c^{*}\left(\langle F\left(a_{0}\right),b-a_{0}\rangle +\text{qri}\;C\right)+\epsilon . \end{array}$$

Since the inequality holds for each *ϵ* > 0, we deduce that

(49) $$\begin{array}{c} c^{*}\left(\langle F\left(a_{0}\right),b-a_{0}\rangle +\text{qri}\;C\right)\geq 0, \end{array}$$

whence, by using Lemma 8, we get

(50) $$\begin{array}{c} \langle F\left(a_{0}\right),b-a_{0}\rangle \notin -C\setminus \left\{0\right\}-\text{qri}\;C\subseteq -\text{qri}\;C, \end{array}$$

which completes the proof.
